# mRNA Galsomes Vaccine Protects Budgerigars Against Virulent *Chlamydia psittaci* Challenge

**DOI:** 10.3390/vaccines13020206

**Published:** 2025-02-19

**Authors:** Anne De Meyst, Joeri Van Mieghem, Koen Chiers, Koen Raemdonck, Rein Verbeke, Ine Lentacker, Daisy Vanrompay

**Affiliations:** 1 Department of Animal Sciences and Aquatic Ecology, Faculty of Bioscience Engineering, Ghent University, 9000 Ghent, Belgium; anne.demeyst@ugent.be (A.D.M.); jvanmieghem@3-14.com (J.V.M.); 2 Department of Pathobiology, Pharmacology and Zoological Medicine, Faculty of Veterinary Medicine, Ghent University, 9820 Merelbeke, Belgium; koen.chiers@ugent.be; 3 Ghent Research Group on Nanomedicines, Department of Pharmaceutics, Faculty of Pharmaceutical Sciences, Ghent University, 9000 Ghent, Belgium; koen.raemdonck@ugent.be (K.R.); rein.verbeke@ugent.be (R.V.); ine.lentacker@ugent.be (I.L.)

**Keywords:** mRNA, vaccine, adjuvant, *Chlamydia psittaci*, zoonosis, avian

## Abstract

Background/Objectives: *Chlamydia (C.) psittaci* is an avian respiratory pathogen that regularly infects budgerigars (*Melopsittacus undulatus*) and is a known zoonosis. This study aimed to evaluate the efficacy of a nucleoside-modified mRNA vaccine formulated in lipid nanoparticles (LNPs), either with (mRNA Galsomes) or without (mRNA LNPs) the glycolipid antigen α-Galactosylceramide, in protecting budgerigars against *C. psittaci* genotype A infection. Methods: Three groups of eight budgerigars received two intramuscular vaccinations with PBS, mRNA LNPs or mRNA Galsomes, and were subsequently challenged via aerosol with the *C. psittaci* genotype A strain 90/1051. Vaccine efficacy was assessed over 14 days post challenge by monitoring clinical signs, macroscopic and microscopic lesions, pathogen excretion and chlamydial burden in organs. Antibody levels were evaluated at baseline, after vaccination and post challenge. Results: Both mRNA LNPs and mRNA Galsomes induced significant serum antibody responses post booster. Vaccination significantly reduced clinical signs, chlamydial burden in the lungs and macroscopic lesions in conjunctiva, conchae, lungs and thoracic airsacs, compared to controls. Additionally, mRNA Galsomes-treated birds showed a significantly reduced lung inflammation and fewer macroscopic lesions in abdominal airsacs and liver, compared to non-vaccinated animals. These animals also experienced a significantly lower chlamydial burden in the spleen, fewer clinical signs at day 11 and fewer fecal shedding at day 14 post challenge, compared to mRNA LNP-treated animals. Conclusions: This study demonstrated that mRNA vaccination confers partial protection against *C. psittaci* in budgerigars, with mRNA Galsomes appearing to provide enhanced efficacy. However, the absence of species-specific reagents for assessing cellular immunity in *Psittaciformes* limits a comprehensive understanding of vaccine-induced protection. The development of psittacine-specific T cell markers and cytokine assays is necessary to further elucidate immune mechanisms and optimize vaccine formulations.

## 1. Introduction

*Chlamydia psittaci* (*C. psittaci*) is a Gram-negative bacterium, classified within the *Chlamydiaceae* family. This obligate intracellular bacterium can invade over 465 bird species, including domestic, wild and pet birds. A systematic review by Sukon and colleagues in 2021, incorporating data from 74 epidemiological studies, estimated that 19.5% (95% CI, 16.3–23.1%) of all birds worldwide are *Chlamydia*-positive, with *C. psittaci* the predominantly reported agent [1]. Infected birds may remain asymptomatic or exhibit respiratory symptoms, including dyspnea, lethargy, conjunctivitis, rhinitis and diarrhea, with severe cases potentially leading to systemic multiple-organ failure and death. *C. psittaci* is mostly transmitted through inhalation of infected aerosols and is a well-known zoonosis. When transmitted to humans, it may cause flu-like illness to severe pneumonia, named psittacosis [2].

*C. psittaci* is classified into genotypes based on variations in the *OmpA* gene, encoding the major outer membrane protein (MOMP). Fifteen genotypes are already defined, with each showing their own host predilection, virulence and zoonotic potential. Genotype A is primarily associated with psittacine birds and is highly virulent. Other genotypes (B–F, E/B, 1V, Mat116, etc.) are linked to infections in pigeons, ducks, turkeys and other avian species. While all *C. psittaci* genotypes are believed to be transmittable to humans, some are more prevalent than others. Psittacine birds are the primary source of human infections, with genotype A the predominantly reported genotype in hospitalized psittacosis patients. Consequently, psittacine bird keepers or caretakers are a known risk population [3,4].

Current treatments for *Chlamydia* infections in birds and humans rely on tetracyclines and macrolides; however, rising antimicrobial resistance necessitates alternative approaches, especially for this zoonotic pathogen [5]. Vaccination is a prophylactic measure to protect birds against disease and prevent transmission to humans, yet no commercial *C. psittaci* vaccine is available. Early vaccine development focused on inactivated whole pathogens but yielded only moderate protection and raised safety concerns due to *C. psittaci*’s biosafety level 3 classification [6,7]. Efforts to develop DNA vaccines targeting MOMP demonstrated partial efficacy, with strategies such as DNA::MOMP polyplex formulation improving, but not achieving full protection [8]. More recent approaches have explored the use of (1) alternative antigens (e.g., plasmid or polymorphic membrane proteins) [9,10], (2) alternative vaccine platforms (e.g., recombinant or inactivated vaccines) [11,12] and (3) alternative administration routes (e.g., systemic, mucosal, or combined approaches) [13], though complete protection has remained elusive.

mRNA vaccination is a promising innovative vaccine technology, valued for its ease of production, strong safety profile and effectiveness in generating antigen-specific adaptive immunity. However, their use in avian species and other animals remains largely unexplored [14]. Therefore, this study evaluated the efficacy of a nucleoside-modified mRNA vaccine encapsulated in a lipid nanoparticle (LNP) for efficient intracellular delivery and protection against nucleases. The LNP contained the ionizable lipidoid C12-200, together with the helper lipid 1,2-dioleoyl-sn-glycero-3-phosphocholine (DOPC), cholesterol and PEGylated lipids to create a steric barrier. The mRNA vaccine encoded a polyepitope of *C. psittaci* MOMP and was formulated with and without inclusion of the glycolipid antigen α-Galactosylceramide (αGalcer).

A polyepitope of *C. psittaci* MOMP genotype A was selected as the antigen, based on the data of multiple immunization studies demonstrating its protective potential [15,16]. This polyepitope included B cell epitopes, CD4+ Th1, CD4+ Th2 and CD8+ T cell epitopes. While the inclusion of CD4+ Th2 and CD8+ T cell epitopes has been associated with increased pathology in murine and bovine models, studies in chickens demonstrated that this combination can confer superior protection against *C. psittaci* challenge compared to full recombinant MOMP, a combination of B cell and CD4+ Th2 epitopes, or a combination of CD4+ Th1 and CD8+ T cell epitopes alone [17,18,19].

The adjuvant αGalCer was included in the vaccine due to its ability to activate invariant Natural Killer T (iNKT) cells in mammals. This iNKT agonist binds to CD1d, an MHC-I-like molecule on dendritic cells and other antigen-presenting cells, triggering a rapid release of both CD4+ Th1 and CD4+ Th2 cytokines. Additionally, αGalCer-stimulated iNKT cells enhance CD8+ T cell and NK cell activation, leading to the production of protective IFN-γ. This broadens the immune response and strengthens cellular immunity, as demonstrated in both prophylactic and therapeutic applications in mice [20,21]. Given that *Chlamydia* immunity relies on a Th1-mediated cellular response driven by IFN-γ-secreting CD4+ and CD8+ T cells, αGalCer is a promising adjuvant candidate [22]. Furthermore, NKT cells were previously shown to have a protective role in *Chlamydia* infections. In *C. pneumoniae*-infected mice, for example, activated NKT cells upregulated costimulatory molecules and cytokines in lung dendritic cells, promoting a protective Th1-oriented response. On the other hand, NKT cells have also been implicated in increased susceptibility to *C. muridarum* infection, as they can produce IL-4 and activate Th2 cells, highlighting that closely related pathogens can induce distinct NKT subsets [23,24].

Although the incorporation of αGalCer in a *Chlamydia* vaccine seems promising, to date, the role of NKT cells in *C. psittaci* infections has not been investigated. Furthermore, the effectiveness of αGalCer as an iNKT agonist in birds remains uncertain, given that avian CD1 molecules possess distinct binding pockets compared to their mammalian counterparts [25]. Lastly, iNKT cell populations have not been characterized in *Psittaciformes*, rendering it uncertain whether αGalCer will be able to activate this subset of cells.

Nevertheless, we hypothesize that an mRNA vaccine will provide protection against *C. psittaci* infection in budgerigars and that the incorporation of αGalcer will provide superior protection compared to a non-adjuvanted mRNA vaccine.

Our findings show that both mRNA vaccines elicited high serum antibody titers in budgerigars and provided partial protection against *C. psittaci* challenge. The mRNA vaccine with αGalCer appeared to offer greater protection compared to mRNA LNPs without αGalCer. Whether this effect is due to enhanced cellular immunity following iNKT activation remains unclear, as cellular immunity was not assessed due to the lack of *Psittaciformes*-specific immunological tools.

## 2. Materials and Methods

### 2.1. Vaccine Engineering

The mRNA platform was constructed by incorporating the following elements in the multiple cloning site of the minimalistic picoZ vector [26], in a 5′ to 3′ order: a T7 polymerase promotor, a Cleancap^®^ AG trimer, a minimalistic 5′ untranslated region (UTR) [27], a combined 3′ UTR region of AES and mtRNR1 [28], and a segmented polyA-tail of 100 adenosines [29]. Between the 5′ and 3′ UTR, a polyepitope of the *ompA* gene of *C. psittaci* genotype A (accession number: AF269281.1) was inserted after codon optimization for use in birds. This polyepitope included B-cell epitopes, CD8+ T cell epitopes, CD4+ Th1, and Th2 epitopes, as described in [17].

mRNA production was performed by in vitro transcription from a DNA template. First, the picoZ plasmid was heat shock transformed into *Escherichia coli* DH5α (Thermo Scientific, Illkrich, France). After transformant selection and successive culture, the plasmid was purified with the PureLink™ Expi Endotoxin-Free Mega Plasmid Purification Kit (Invitrogen, Carlsbad, CA, USA). Next, the plasmid was linearized using the Sce-I restriction enzyme (New England Biolabs Inc., Leiden, The Netherlands; 10 units/µg DNA) by overnight incubation at 37 °C, and the product was subsequently purified using a Wizard SV Gel and polymerase chain reaction (PCR) Clean-Up System (Promega, Leiden, The Netherlands). The linearized plasmid was subsequently transcribed into mRNA using the HiScribe T7 High Yield RNA Synthesis Kit (New England Biolabs Inc.), and the mRNA was purified using the Monarch RNA Cleanup kit (New England Biolabs Inc.). Both kits were executed according to the manufacturer’s instructions. During transcription, mRNA was capped with the CleanCap^®^ AG kit (Trilink Biotechnologies, Boechout, Belgium), and the modified base methyl-1-pseudoridine (Trilink Biotechnologies) was incorporated as a replacement for uridine. Afterwards, mRNA was purified with cellulose (Merck Life Science, Hoeilaart, Belgium) to remove residual dsRNA contaminants as described by Baiersdörfer et al. (2019) [30]. Lastly, mRNA integrity was assessed with bleach gel electrophoresis according to Aranda et al. (2012) [31]. A clear 1386 bp band without smearing confirmed the mRNA’s high quality and the absence of nuclease degradation.

LNPs were freshly prepared for each immunization with a T junction mixer. Therefore, C12-200, cholesterol, 1,2-dimyristoyl-rac-glycero-3-methoxypolyethylene glycol-2000 (DMG-PEG-2K), and DOPC were purchased at MedChemExpress (Sollentuna, Sweden) and dissolved in 100% ethanol at respective molar ratios of 35 mol%, 46.5 mol%, 2.5 mol%, and 16 mol%. The mRNA was mixed with a 25 mM sodium acetate buffer (pH 4; Thermo Scientific Chemicals, Illkrich, France). Next, mRNA and lipids were mixed at a flow rate of 20 mL/min (15 mL/min for RNA and 5 mL/min for lipids) with a C12-200 to mRNA weight ratio of 20. The adjuvant αGalcer was synthesized in the Laboratory of Medicinal Chemistry, Ghent University, as described before [21], and incorporated in the LNP by addition to the lipid mixture at a molar ratio of 0.02 mol%. Subsequently, the LNPs were dialyzed overnight in Slide-A-Lyzer™ Dialysis Cassettes using PBS as a buffer (Thermo Scientific; 20K MWCO) and concentrated afterwards using Amicon^®^ Ultra Centrifugal Filters (Merck Life Science; 10 kDa MWCO). Lastly, LNPs were filtered with 0.20 µm Acrodisc 25 mm Syringe filters (Merck Life Science) and RNA concentration was determined with the RediPlate™ 96 RiboGreen™ RNA Quantitation Kit (Invitrogen). LNP zeta potential and particle size were measured with a Zetasizer Nano ZS (Malvern Instruments, Hoeilaart, Belgium). Ten micrograms of each formulation was diluted in 20 mM HEPES (pH 7.4; Thermo Scientific Chemicals) and transferred to a disposable folded capillary cell (DTS1070). Each measurement was conducted in triplicate at a temperature set to 25 °C.

### 2.2. Parakeet Selection and Experimental Set-Up

**Figure 1:**
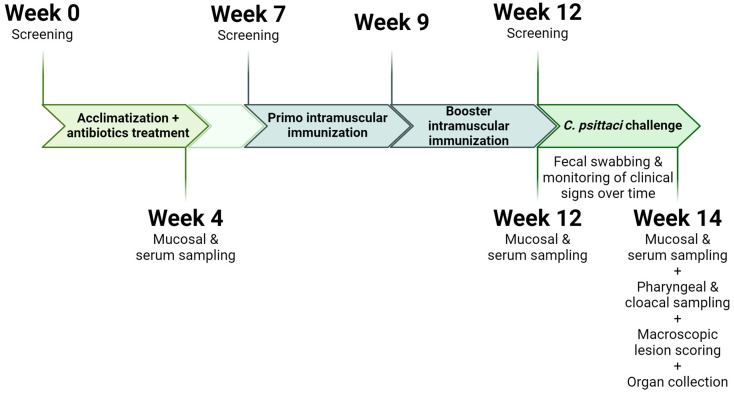
Immunization and sample collection scheme of the efficacy trial (created with Biorender.com).

Twenty-four budgerigars (*Melopsittacus undulatus*) were obtained from a breeding facility in East-Flanders. Since SPF *Psittaciformes* are not available, all animals were tested for the presence of *C. psittaci* DNA upon arrival. Therefore, cloacal and pharyngeal swabs (Minitip FLOQSwabs^®^, Novolab, Geraardsbergen, Belgium) were collected in 0.4 mL DNA stabilization buffer (Merck Life Science). After shaking for 1 h at 4 °C, DNA was extracted with the QIAamp^®^ DNA mini kit (Qiagen, Antwerp, Belgium), followed by a *C. psittaci*-specific PCR [32]. This PCR detects all *C. psittaci* genotypes with a sensitivity of 1 inclusion forming unit/reaction. Subsequently, *Chlamydia*-negative animals received weekly intramuscular treatments (*musculus pectoralis major*) of Vibramycin SF^®^ (Pfizer, Puurs, Belgium) at a dosage of 100 mg doxycycline/kg body weight, over a span of four weeks, as a precaution to exclude carrier birds [33]. After a three-week wash-out period, a second screening was performed and *C. psittaci*-negative birds were randomly assigned to three groups (control, mRNA LNPs, and mRNA Galsomes), each consisting of 4 bird pairs (*n*= 8). All budgerigars were immunized intramuscularly with PBS, 10 µg mRNA LNPs, or 10 µg mRNA Galsomes (50 µL injection volume) respectively. We opted for intramuscular vaccination as it is one of the most convenient parenteral administration routes and has been demonstrated to effectively induce a protective immune response against *C. psittaci* before [16]. A 10 µg dose was selected based on its optimal efficacy in modified mRNA vaccination studies in mice, which have a metabolic weight comparable to budgerigars [20,34]. Groups received a primo vaccination, followed by a booster vaccination two weeks later. Three weeks post booster, a last screening was performed, and all animals were challenged by aerosol (Cirrus^TM^ Nebulizer, Intersurgical, Wokingham, Berkshire, UK) with 10^8^ TCID_50_ of the virulent *C. psittaci* strain 90/1051. This strain originated from a decedent Blue-fronted amazon and was typed as genotype A through whole genome Nanopore sequencing (unpublished data; [35,36]). Animals were euthanized 14 days post challenge. The immunization and sample collection scheme are illustrated in Figure 1.

During the animal trial, the twelve pairs of female and male budgerigars were randomly assigned to twelve separate cages. Birds within each cage were subjected to the same treatment, and all cages were placed next to each other in the same room. The animals were provided with food (comprising budgerigar seed mix, millet, grit and coral mix, mineral mix, and fruit sticks) and water ad libitum. From the experimental infection onward, all cages were placed in three isolators with a negative pressure (IM1500, Montair, Sevenum, The Netherlands), with each group housed in an individual isolator to prevent cross-infection.

### 2.3. Sampling of Birds

Blood samples were collected for the quantification of *Chlamydia*-specific serum antibody titers during initial screening (week 4), before challenge (week 12), and at euthanasia (week 14), by performing a venipuncture on the cutaneous ulnar vein (*v. ulnaris*). Blood samples were stored at room temperature overnight and serum was collected by centrifugation at 10,000× *g* for 10 min (4 °C) and subsequently stored at −80 °C until further analysis. Simultaneously, pharyngeal swabs were collected in 0.4 mL cOmplete™ protease inhibitor cocktail (Merck Life Science) for the quantification of *Chlamydia*-specific mucosal antibodies and stored at −80 °C until further analysis. Post challenge, all budgerigars were monitored daily for clinical signs. Fecal excretion was evaluated at day 3, 5, 8, 12, and 14 post challenge, by collecting swabs from freshly deposited feces on the cage floors. Further, cloacal and pharyngeal swabs were collected at euthanasia, 14 days post challenge. Swabs were stored in chlamydial transport medium and stored at −80 °C until later use. At euthanasia, macroscopic lesions were scored by a veterinarian in conjunctivae, conchae, trachea, thoracic and abdominal airsacs, lungs, pericardium, spleen, liver, kidneys, gut, and pectoral muscle. Further, lung, liver, kidney, spleen, airsacs, and pectoral muscle were collected in 10% phosphate buffered formalin (Sigma-Aldrich, Hoeilaart, Belgium) and methylcellulose (Thermo Scientific Chemicals). Organs in methylcellulose were stored at −80 °C for the production of cryostat tissue sections and subsequent quantification of *Chlamydia*. Organs in formalin were stored at room temperature for histopathological analysis. All samples were blinded prior to storage.

### 2.4. Antibody Response

An indirect enzyme-linked immunosorbent assay (ELISA) was developed to measure specific anti-*C. psittaci* antibodies in budgerigar serum and mucosal swabs, based on [37]. Since anti-budgerigar antibodies are not commercially available, cross-reaction between goat anti-chicken immunoglobulin (Ig)Y(H+L)-Biotin (Southern Biotech, Cambridge, UK) and budgerigar serum was confirmed first. Therefore, immunoglobulins were purified from budgerigar serum with a Pierce™ Thiophilic Adsorption Kit (Thermo Scientific), after which cross-reaction was confirmed with Western Blot. All serum samples were kaolin-pretreated (Merck Life Science) to reduce background noise in the assay [38]. Mucosal swabs were not pretreated.

To detect anti-*C. psittaci* antibodies, 96 well ELISA MaxiSorp plates (Thermo Scientific) were coated for 3 h at 37 °C with 100 µL UV-inactivated 10^6^ TCID_50_ of *C. psittaci* strain 90/1051 (genotype A). Subsequently, wells were blocked overnight (4 °C) with 200 µL PBS and 5% bovine serum albumin (BSA; Sigma-Aldrich). One hundred microliters of two-fold diluted (1/50 until 1/12,800) pretreated serum was added to the blocked plates. All dilutions were made in dilution liquid (DL), containing PBS, 3% BSA and 0.05% Tween 20 (Sigma-Aldrich). Alternatively, 100 µL mucosal swab solution was added, diluted 1/10 in DL. All samples were tested in duplicate. After a 1 h incubation (37 °C), 100 µL goat anti-chicken IgY(H+L)-Biotin, diluted 1/250 in DL, was added and incubated at 37 °C for an additional hour. Next, Streptavidin-PolyHRP_20_ (SDT GmbH, Baesweiler, Germany) was diluted 1/2000 in DL and 100 µL conjugate was added to each well. At last, after an incubation of 1 h at 37 °C, 50 µL of a 1:1 mixture of ABTS:H_2_O_2_ (ABTS^®^ 2-Component Microwell Peroxidase Substrate Kit, SeraCare Life Sciences, Nazareth, Belgium) was brought into each well. Between each step, plates were manually washed four times with PBS and 0.05% Tween 20. Absorbance was measured at a wavelength of 405 nm (A_405_) each 15 min, after shaking the plate for 5 s. Samples were considered positive if their average A_405_ exceeded the average A_405_ of the negative controls added with three times its standard deviation. Anti-*C. psittaci* Ig titers were presented as the reciprocal of the highest serum dilution at which samples were considered positive.

### 2.5. Chlamydia Excretion

Fecal, cloacal and pharyngeal samples were analyzed for the presence of live *Chlamydia*. Briefly, samples were inoculated onto Buffalo Green Monkey cells (BGM, Cytion catalog number 302158, Eppelheim, Germany) [39] and incubated afterwards at 37 °C for six days. Next, *Chlamydia* was visualized with Imagen™ direct immunofluorescence staining after bleaching the cells under a LED light source (20,000 LUX) for 36 h at 4 °C in antigen unmasking solution (2BScientific, Oxfordshire, UK). For each cage or bird pair (in case of fecal samples) and each bird (in case of pharyngeal and cloacal samples), the complete slide was inspected and scored as follows: (0) no *Chlamydia* present, (1) few EBs visible (<20), (2) *Chlamydia* present in <10% of the microscopic fields, (3) *Chlamydia* present in 10–30% of the microscopic fields, (4) *Chlamydia* present in 10–30% of the microscopic fields with large EB islets and/or inclusions, (5) *Chlamydia* present in 30–50% of the microscopic fields, (6) *Chlamydia* present in >50% of the microscopic fields and (7) *Chlamydia* present in >50% of the microscopic fields with multiple mini- and midi inclusions.

### 2.6. Macroscopic and Microscopic Lesions

Macroscopic lesions were scored for each organ individually at euthanasia by a veterinary pathologist, according to Supplementary Materials Table S1 and as described by Vanrompay et al. (1994) [40]. In brief, a score of 1 was assigned when mild lesions were visible, a score of 2 represented moderate lesions, and a score of 3 denoted severe or advanced pathology. A score of 0 indicated the absence of lesions. Histopathology was performed on 5 µm thick paraffin-embedded sections following hematoxylin (Merck life science) and eosin (Merck life science) staining. A veterinary pathologist assigned histological grades (ranging from 0, minimal change, to 5, severe change) to specific histological observations characteristic for inflammation or pathology. For each bird, a final histological score was calculated for both categories by summing up the grades from the individual observations [40]. All histological observations are detailed in Supplementary Materials Table S2.

### 2.7. Chlamydia Presence in Bird Tissues

Cryostat tissue sections of different organs were cut at 5 µm thick and air-dried onto precoated poly-L-lysine (Sigma-Aldrich) microscopic slides. Sections were fixed in pre-cooled acetone (Merck life science) for 15 min at −20 °C, air-dried for 1 h and washed with PBS twice. Next, sections were placed on a LED light source (20,000 LUX) for 36 h at 4 °C in antigen unmasking solution (2BScientific). Bleached sections were inspected using Imagen™ direct immunofluorescence staining in duplicate. The presence of *Chlamydia* antigens was scored as described above for swab cultures.

### 2.8. Statistics

The statistical methodology was based on Morgan (2017) [41]. Briefly, normality was evaluated using a QQ plot and homoscedasticity was assumed if the ratio of the largest to the smallest variance did not exceed three. For normally distributed and homoscedastic data, group means were compared using an ANOVA test, while a Welch ANOVA test was applied in cases of heteroscedasticity. If normality could not be assumed, a Kruskal–Wallis test was used for homoscedastic data, whereas permutation tests were employed for heteroscedastic data. Statistical testing was performed with GraphPad Prism 8.0.1, Rstudio 4.2.3, or SPSS Statistics 28, with a significance level of 0.05. Statistical significance was indicated as (*) for *p* < 0.05, (**) for *p* < 0.01, or (***) for *p* < 0.001, unless otherwise detailed in figure or table captions. No data points were excluded during any of the analyses.

## 3. Results

### 3.1. Vaccine Engineering

Nucleoside-modified mRNA was produced and its quality was assessed with bleach gel electrophoresis (Supplementary Materials Figure S1). Afterwards, the mRNA was formulated in LNPs composed of the ionizable lipid C12:200, DOPC, cholesterol, and DMG-PEG, with and without inclusion of the iNKT agonist αGalcer. Particle size and zeta potential were measured in HEPES buffer (Table 1). All particles were nearly neutral with an average zetapotential of 7.7 mV and z-average size of 111 nm.

### 3.2. Antibody Response

**Figure 2:**
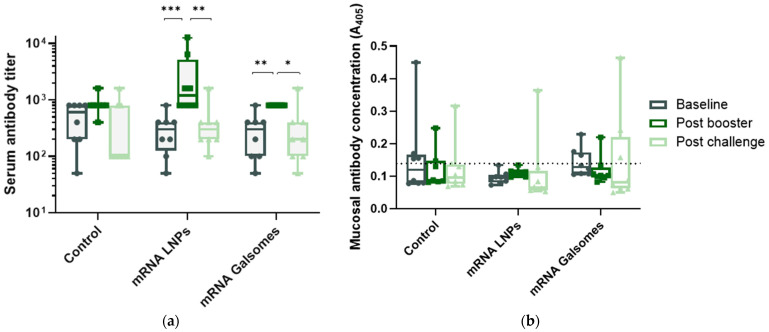
(**a**) Binding anti-C. psittaci serum antibody titers expressed as the reciprocal of the last serum dilution with an absorbance exceeding the cut-off value, calculated as Ave(neg) + 3 × SD(neg). (**b**) Binding anti-C. psittaci mucosal antibodies expressed as absorbance values at 405 nm. The dashed line represents the cut-off value. Differences between timepoints were determined with permutation tests and indicated as (*) for *p* < 0.05, (**) for *p* < 0.01, or (***) for *p* < 0.001.

The production of binding antibodies following vaccination and challenge was determined with a C. psittaci-specific ELISA and is shown in Figure 2. All animals exhibited a baseline serum antibody titer of at least 50 during initial screening (week 4), indicating prior exposure to C. psittaci. Nevertheless, there were no significant differences in baseline serum antibody titers among the treatment groups, as confirmed by a permutation test. Serum antibody titers were significantly higher than baseline levels three weeks post booster vaccination with mRNA LNPs (*p* < 0.001) and mRNA Galsomes (*p* = 0.006). Two weeks post challenge, antibody titers reverted to their initial baseline level across all groups, including the control group. The highest serum antibody titers were recorded in animals administered with mRNA LNPs, three weeks post booster vaccination. At that timepoint, two animals receiving mRNA LNPs reached exceptionally high serum antibody titers of 12,800 and 6400, respectively.

Mucosal antibody levels exhibited no significant variations across different timepoints (Figure 2). Noteworthy, one bird treated with mRNA LNPs and one bird treated with mRNA Galsomes displayed an increase in mucosal antibodies post challenge. Additionally, one bird in the control group maintained a higher mucosal antibody level throughout the entire animal study.

### 3.3. Clinical Signs

Budgerigars were monitored for clinical signs until 14 days post challenge. During this period, birds from the control group experienced clinical symptoms such as squinting or continuous blinking with eyes (conjunctivitis; 8/8), drowsiness, anorexia, ruffled or puffed-up feathers, a hunched head, or apathy (lethargy; 8/8), labored breathing, open beak breathing, gasping, or wings held away from the body (dyspnea; 8/8), and yellow green watery droppings (diarrhea; 4/8). Animals that received the mRNA LNPs vaccine showed symptoms of lethargy (4/8), dyspnea (4/8), and diarrhea (2/8), whereas animals receiving the mRNA Galsomes vaccine suffered from lethargy (2/8), dyspnea (3/8), and diarrhea (2/8).

**Figure 3:**
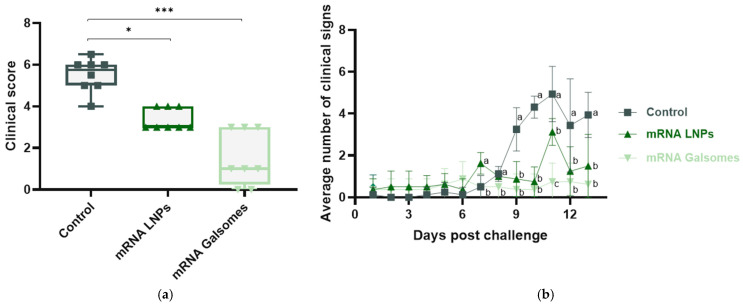
(**a**) Individual clinical scores per bird, calculated as a sum of points, with 1 point assigned for each distinct clinical sign observed throughout the entire infection and 1.5 points for particularly severe symptoms. (**b**) Average number of clinical signs per treatment group over time. For **a**, differences between treatment groups were determined with permutation tests and indicated as (*) for *p* < 0.05, (**) for *p* < 0.01, or (***) for *p* < 0.001. For **b**, differences between treatment groups were determined with Kruskal–Wallis tests and indicated with a different letter when significant.

Each bird was assigned a clinical score, calculated as a sum of points, with 1 point for each distinct clinical sign observed throughout the entire infection and 1.5 points for particularly severe symptoms. These scores are illustrated in Figure 3a, together with the average number of clinical signs over time in Figure 3b. Figure 3a shows that both mRNA LNP- and mRNA Galsomes-treated animals experienced significantly fewer clinical signs as the control group (*p* = 0.020 and *p* < 0.001, respectively). There was no significant difference in clinical score between both treatment groups. The highest number of clinical signs was observed on day 11, after which they gradually declined. On day 11, the control group exhibited significantly more clinical signs compared to both mRNA LNP- (*p* = 0.001) and mRNA Galsomes-treated animals (*p* < 0.001). Additionally, mRNA Galsomes-treated animals showed significantly fewer clinical signs than mRNA LNP-treated animals (*p* < 0.001).

### 3.4. Chlamydia Excretion

**Figure 4:**
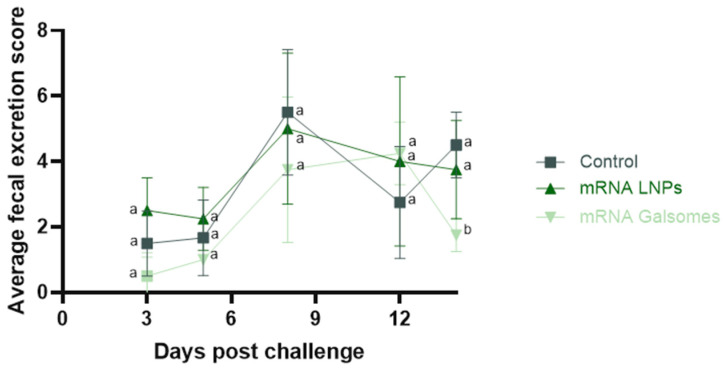
Average fecal excretion per cage or bird pair in function of time. Differences between treatment groups were determined with Kruskal–Wallis tests and post hoc Dunn tests and indicated with a different letter when significant.

Fourteen days after the challenge, all animals excreted viable Chlamydia bacteria. Pharyngeal and cloacal excretion of C. psittaci was lower in both immunized groups, albeit not significantly different from the excretion in the non-vaccinated control group (Table 2). Fecal excretion varied among time in all groups (Figure 4) and was maximal 8 days post challenge for the control group and mRNA LNPs group. In the mRNA Galsomes group, excretion was maximal 12 days post challenge. A significant difference in excretion levels between groups was observed only on day 14. The mRNA Galsomes-treated group excreted significantly less Chlamydia compared to both the mRNA LNP-treated animals (*p* = 0.044) and the control group (*p* = 0.015).

### 3.5. Macroscopic and Microscopic Lesions

Following lesions were observed in the control group at euthanasia: bilateral congestion of conjunctiva (8/8) and conchae (6/8), diffuse opacity (4/8) or focal fibrin deposits (1/8) in airsacs, bilateral lung congestion (7/8) and presence of pneumonic foci (5/8), enlarged spleen (2/8), enlarged liver (5/8), and kidney congestion (2/8). The presence of macroscopic lesions in both respiratory and non-respiratory organs suggests that the infection spread systemically in 6/8 control birds. mRNA LNP-vaccinated animals suffered from congestion in the lungs (4/8), focal fibrin deposits in airsacs (1/8), enlarged spleen (3/8), and enlarged liver (1/8). The most lesions were found in respiratory organs and only in three birds the infection spread to other organs. In the group receiving a mRNA Galsomes vaccine, only one bird exhibited lung congestion. Remarkably, none of the birds displayed discernible damage at the site of vaccination, the pectoral muscle. Pictures of fibrin deposits in airsacs, congestion of the conjunctiva and pneumonic foci in control birds can be found in Supplementary Materials Figure S2.

The macroscopic lesion scores per organ are summarized in Table 3. A significantly higher score was attributed to the conjunctiva (*p* < 0.001) and conchae (*p* = 0.003) of control birds, compared to treated animals. Additionally, the lungs and thoracic airsacs received significantly lower scores in birds vaccinated with mRNA LNPs (*p* = 0.006 and *p* = 0.041, respectively) and mRNA Galsomes (*p* = 0.003 and *p* = 0.003, respectively), compared to controls. Birds vaccinated with mRNA Galsomes exhibited significantly fewer macroscopic lesions in abdominal airsacs (*p* = 0.010) and liver (*p* = 0.011), compared to the control group. Remarkably, despite all animals being infected with a dose of 10^8^ TCID_50_ of C. psittaci, the control group exhibited relatively modest macroscopic lesion scores. Only three organs obtained an average score surpassing 1 point, with the highest possible score being 3 points.

Histopathological data were obtained 14 days post challenge. Inflammation was characterized by factors such as mononuclear and heterophil cell infiltration and congestion, whereas pathology was characterized by occurrences of necrosis and fibrinous exudation. The final histological scores can be found in Table 4 and are further detailed in Supplementary Materials Table S2. As per the assessment by the pathologist, the examined organs displayed only mild to moderate lesions. There was no evidence of severe damage in any of the organs within any of the groups. The lower airways, spleen and pectoral muscle (immunization site) displayed no signs of pathology across all groups. When comparing control animals to treated animals, it was observed that vaccination did not significantly lower pathology in any organ.

**Figure 5:**
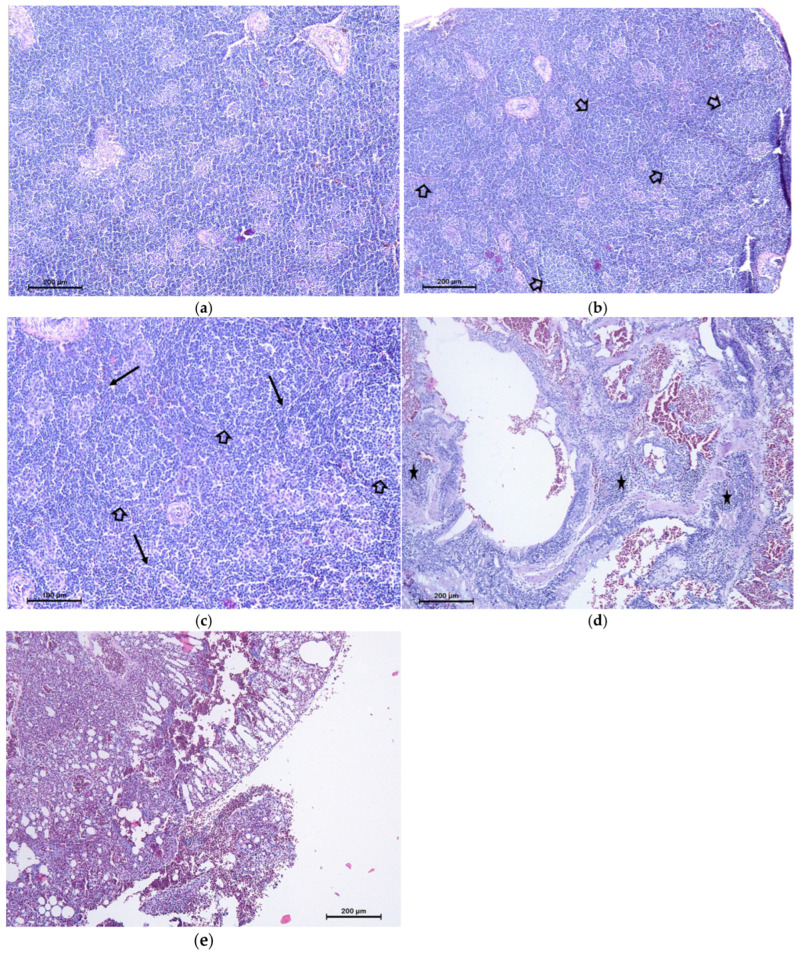
Haematoxylin eosin stain on tissue organs demonstrating (**a**) a quiescent spleen of a control animal; (**b**) an activated spleen of a vaccinated animal; (**c**) a detail of Sweigger-Seidel sheaths and follicles present in the spleen of a vaccinated animal; (**d**) lymphoid infiltration in the lung epithelial layer of a control animal; and (**e**) the absence of lymphoid infiltration in vaccinated animals. Stars indicate lymphoid infiltration, visible as small blue lymphocytes within the epithelial layers. Empty arrows point to lymphoid follicles, which appear as circular structures filled with small blue lymphocytes. Full arrows highlight Sweigger-Seidel sheaths, which are spindle-shaped, thickened arterioles surrounded by sheaths of reticular cells and ellipsoid-associated cells.

As the spleen is the most important secondary lymphoid organ in birds, immune activation was examined more closely in these organs. The peri-arterial lymphoid sheaths of the white pulp were pronounced in 3/8 (37.5%) and 5/8 (62.5%) birds of mRNA LNP- and mRNA Galsomes-vaccinated animals, respectively. This phenomenon was not observed in control birds (Figure 5a,b). In addition, pronounced ellipsoids of reticular cells (Schweigger Seidel sheaths; Figure 5c) were visible in the white pulp of 2/8 (25%) birds of both vaccinated groups, whereas this was not the case for the control birds. Macrophages could be observed between the reticular cells.

Inflammation was primarily observed in the upper and lower airways. Animals receiving mRNA Galsomes exhibited significantly fewer signs of inflammation in upper airways compared to controls (*p* = 0.019). Lymphoid infiltration was observed in the lung epithelial layer of control animals, whereas this was not the case in vaccinated animals (Figure 5d,e). Interestingly, mRNA Galsomes-treated animals exhibited more microscopic lesions of inflammation in both the liver, spleen, and kidney, compared to the control group, albeit also not significantly.

### 3.6. Chlamydia Presence in Tissue Organs

The presence of Chlamydia was scored in both respiratory organs (airsacs and lungs) and non-respiratory organs (spleen, liver, kidney) (Table 5). Chlamydia was detected in all organs except in the spleens of animals vaccinated with mRNA Galsomes, leading to a significantly different score between animals receiving mRNA LNPs and mRNA Galsomes (*p* = 0.014). Significantly fewer Chlamydia were present in the lungs of both mRNA LNP-treated (*p* = 0.004) and mRNA Galsomes-treated animals (*p* < 0.001), compared to controls. Additionally, mRNA Galsomes-treated animals displayed significantly lower Chlamydia levels in the liver, compared to the control group (*p* = 0.024). No significant differences were observed between the three groups in airsacs and kidney.

## 4. Discussion

*C. psittaci* is an obligate intracellular and biphasic bacterium that infects the respiratory system of birds and humans. Psittacine birds are endemically infected with virulent *C. psittaci* strains and frequently transmit the pathogen to humans. Regulatory vaccination could limit the circulation of *Chlamydia* strains in these birds and reduce zoonotic transmission, but no vaccine is currently available [33]. In this study, an mRNA vaccine was developed and evaluated for its potential to protect budgerigars against *C. psittaci* infection. The possibility of using mRNA vaccines in birds was recently demonstrated when a self-amplifying mRNA reporter vaccine was successfully delivered in broiler chicken explant models and in ovo [42]. However, this is the first study evaluating the efficacy of an avian mRNA vaccination, providing proof-of-concept of its application in birds. Therefore, birds received two mRNA vaccinations, followed by a challenge through aerosol with *C. psittaci* strain 90/1051 (genotype A). Afterwards, clinical signs, macroscopic and microscopic lesions, excretion, and presence of *Chlamydia* in tissue organs were monitored over a period of fourteen days. Additionally, binding antibody levels were monitored before vaccination, after booster vaccination and after challenge. Cellular immune responses could not be examined due to a lack of *Psittacines*-specific T cell markers and cytokine assays. In order to enhance vaccine efficacy, the mRNA was formulated with α-Galactosylceramide (αGalcer)-adjuvanted LNPs (named mRNA Galsomes) and compared to non-adjuvanted LNPs (named mRNA LNPs).

Vaccination with mRNA LNPs generated a partial protection in budgerigars against *C. psittaci*, evident by the significant reduction in clinical signs, a significantly lower incidence of macroscopic lesions in conjunctiva, conchae, lungs, and thoracic airsacs, and significantly fewer *Chlamydia* bacteria in the lungs, compared to controls. Although the first results seem promising, the mRNA LNPs vaccine was not able to completely protect against disease, as *Chlamydia* bacteria were still present in the liver (resulting in microscopic pathological lesions). It seemed that the vaccine was able to provide protection at the entry site of infection (being the mucosal and respiratory tissues), while systemic immunity appeared insufficient. This is rather unexpected as the vaccine was administered parentally (intramuscular vaccination). The exact reason behind this is unclear but may be linked to the very high infection dose that was used to challenge the budgerigars (10^8^ TCID_50_ of a virulent *C. psittaci* strain). This high dose was selected to facilitate monitoring of disease progression in the control group. Since naïve budgerigars were unavailable due to the endemic nature of *C. psittaci*, all birds had pre-existing immunity, with serum antibody titers between 50 and 800 at the start of the trial. A lower challenge dose might not have induced disease, yet even at this high dose, disease progression in control birds remained mild, with limited macroscopic and microscopic lesion scores and low *Chlamydia* burden in organs. However, employing higher challenge concentrations makes it more difficult to achieve significant reduction in disease progression and shedding in vaccinated animals, requiring superior vaccine efficacy. To monitor vaccine efficacy in conditions more closely to field situations, a lower challenge dose should be considered in combination with the use of naïve budgerigars, to prevent prior immune priming from affecting disease progression. Unfortunately, obtaining truly naïve birds is challenging [43,44].

The primary objective of a *C. psittaci* vaccine is to reduce disease severity and pathogen circulation among birds, while mitigating the risk of transmission to humans. However, as previously demonstrated with mRNA vaccines targeting the respiratory SARS-CoV-2, preventing pathogen excretion remains challenging, even when vaccination effectively protects against disease [45,46]. Similarly, in this study, reducing transmission through excretion proved difficult. No significant reduction was observed in pharyngeal or cloacal excretion 14 days post challenge or in fecal excretion over time, although mRNA LNP-vaccinated birds had a lower average excretion score than controls. Even though mRNA vaccination against *C. psittaci* shows promise, it does not yet sufficiently limit excretion to reduce circulation in birds or avoid transmission to humans. Interestingly, previous *C. psittaci* vaccine studies also failed to reduce excretion despite successfully protecting against disease, with only a moderate correlation observed between pharyngeal bacterial load and pathology [22].

Microscopic lesion analysis showed no significant reduction in mRNA LNP-vaccinated birds compared to controls, likely due to the absence of severe lesions in the control group 14 days post challenge. This suggests that the control birds were already recovering, making group differences harder to detect. Assessing pathology at an earlier stage in the infection might have revealed more pronounced lesions, but ethical restrictions prevented the inclusion of additional animals. Interestingly, Schweigger-Seidel sheaths were more pronounced in the spleens of both vaccinated groups. The basic histological structure and function of the Schweigger-Seidel sheath seems to be identical in mammals and birds, but their topographical location is different. In mammals, they are located in the Billroth cords of the red pulp, whereas in birds, they are embedded into the white pulp. Two cell types have been described in the Schweigger-Seidel sheath of chickens: reticular cells possibly with a supporting function and ellipsoid-associated cells (EACs) [47]. Jeurissen et al. (1992) showed that the majority of EACs are macrophages and the rest of the EACs are precursors of interdigitating dendritic cells [48]. Pronounced peri-arterial lymphoid sheaths and pronounced Schweigger-Seidel sheaths thus could reflect an ongoing (protective) immune response in this secondary lymphoid organ.

Based on the results obtained in this efficacy trial, mRNA Galsomes appeared to induce a slightly enhanced protection, as evidenced by significantly less clinical signs at day 11, significantly reduced fecal excretion 14 days post challenge, and significantly decreased *Chlamydia* replication in spleens, compared to mRNA LNP-vaccinated animals. Additionally, mRNA Galsomes significantly limited macroscopic lesions in abdominal airsacs and liver, significantly reduced inflammation in the upper airways and significantly decreased chlamydial burden in the liver, compared to controls—effects not observed with mRNA LNPs. To explore the vaccine’s mechanisms of action, the humoral immune response was examined by monitoring binding antibody levels before vaccination, 21 days post booster, and 14 days post challenge. No significant changes in mucosal antibody production were observed between timepoints, suggesting that systemic vaccination did not effectively induce mucosal humoral immunity. This is in contrast with the observed protection at the pathogen’s entry site, questioning the role of antibodies in protecting against *Chlamydia* infections. On the other hand, in both mRNA LNP- and mRNA Galsomes-treated animals, serum antibody titers significantly increased post booster, indicative of a systemic humoral immune response. Antibody levels between groups were not compared, as the level of prior exposure to *C. psittaci* between groups was not equal, evidenced by a median antibody titer of 800 in control animals, compared to 200 in treated animals. Although mRNA vaccination triggered a humoral immune response, its protective role against *C. psittaci* remains unclear [49]. Earlier vaccine efficacy studies did report a correlation between increased antibody production and protection [15]. However, multiple other studies showed that the presence of binding antibodies does not always correlate with protection, indicating that B cells might contribute to protection in an antibody-independent manner [9,22]. In contrast, cellular immunity, with IFN-γ as key player, is indispensable in combating *Chlamydia* infections [9,22]. Unfortunately, the primary limitation of this study is that cellular immunity was not examined as psittacine-specific T cell markers and cytokine assays are currently unavailable. We hypothesize that, although iNKT cell populations have not been characterized in *Psittaciformes*, they likely recognized αGalCer and enhanced protective immunity against *C. psittaci* by shifting the response toward cellular immunity and linking innate and adaptive immune mechanisms.

To our knowledge, only one previous study attempted to develop a *C. psittaci*-specific vaccine for budgerigars. In that study, birds were vaccinated with a DNA::MOMP vaccine and later challenged with the same *C. psittaci* strain and concentration used in the present study. Vaccination significantly reduced fecal excretion at all examined timepoints (up until day 19 post challenge) and significantly lowered *Chlamydia* replication in all examined organs (lungs, trachea, airsacs, conchae, liver, gut, kidney, and heart). However, half of the DNA-vaccinated budgerigars still suffered from dyspnea, and macroscopic lesions persisted in conchae, lungs, airsacs, and spleen [50]. Compared to this DNA vaccine, mRNA Galsomes provided better protection based on clinical signs and macroscopic lesions, but had only minimal impact on excretion levels and only reduced *Chlamydia* loads in the lungs and liver. Further vaccine optimization is therefore advised. An alternative approach involving mucosal or combined mucosal and systemic administration might lead to a more robust protection, leveraging the strong avian mucosal immune system [51]. Comparative studies already demonstrated that mucosal and combined mucosal and systemic administration can provide superior protection against *C. psittaci* compared to systemic administration alone [13,22]. However, identifying a suitable mucosal route for psittacine birds is challenging: oral administration often induces tolerance and complicates dose monitoring [51,52], while intranasal delivery is hampered by rhinoliths (nose stones) in budgerigars. Eye-drop vaccination, though effective in poultry, is also unsuitable for psittacines due to their small eye surface [53]. One solution to allow mucosal administration is whole-body exposure, where the vaccine is sprayed directly onto the birds. However, this method requires higher doses, significantly increasing the cost of the mRNA vaccine.

## 5. Conclusions

This study evaluates the efficacy of intramuscular immunization with mRNA LNPs and mRNA Galsomes to protect against *C. psittaci* challenge in budgerigars. The mRNA Galsomes vaccine appeared to provide slightly greater protection than mRNA LNPs; however, its precise mechanism of action remains unclear, as cellular immunity could not be assessed. Future studies should be conducted in naïve budgerigars, as prior immune priming may have influenced disease progression in this study. Additionally, incorporating cellular immune assays, such as T cell proliferation and cytokine analysis, would allow a more complete understanding of the generated immune response. Finally, we recommend exploring a combined parenteral and mucosal immunization strategy to enhance antigen-specific immune responses, together with an experimental infective dose which is closer to the one in ‘field’ situations.

## Figures and Tables

**Table 1:** Particle size (Z-average), polydispersity index (PDI) and zetapotential of mRNA LNPs formulated with (mRNA Galsomes) and without (mRNA LNPs) αGalcer in 20 mM HEPES. Values are presented as average ± SD (n = 3).

Treatment	Vaccine	Z-Average (nm)	PDI	Zetapotential (mV)
Primo	mRNA LNPs	119.8 ± 14.2	0.25 ± 0.02	7.0 ± 0.2
vaccination	mRNA Galsomes	104.0 ± 1.4	0.25 ± 0.03	8.0 ± 0.3
Booster	mRNA LNPs	106.5 ± 27.4	0.22 ± 0.01	8.5 ± 1.1
vaccination	mRNA Galsomes	115.4 ± 6.4	0.20 ± 0.01	7.3 ± 0.2

**Table 2:** Cloacal and pharyngeal shedding 14 days post challenge. Values are presented as average score ± SD (% positive animals). Differences between treatment groups were determined with Kruskal–Wallis tests and indicated with a different letter when significant.

Shedding	Control	mRNA LNPs	mRNA Galsomes
Pharyngeal	4.00 ± 2.07 ^a^ (100%)	3.88 ± 1.96 ^a^ (100%)	3.13 ± 1.89 ^a^ (100%)
Cloacal	4.00 ± 2.83 ^a^ (100%)	3.86 ± 2.34 ^a^ (100%)	1.75 ± 1.16 ^a^ (100%)

**Table 3:** Macroscopic lesion score per organ, determined according to Supplementary Materials Table S1. Depending on the severity of the lesions, each organ received a score between 0 (no lesions present) and 3 (severe lesions present). Values are presented as average score ± SD (% positive animals). Differences between treatment groups were determined with Kruskal–Wallis tests and post hoc Dunn tests and indicated with a different letter when significant.

Tissue Organ	Control	mRNA LNPs	mRNA Galsomes
conjunctiva	1.56 ± 0.89 ^a^ (100%)	0.00 ± 0.00 ^b^ (0%)	0.00 ± 0.00 ^b^ (0%)
conchae	1.00 ± 0.76 ^a^ (75%)	0.00 ± 0.00 ^b^ (0%)	0.00 ± 0.00 ^b^ (0%)
trachea	0.00 ± 0.00 ^a^ (0%)	0.00 ± 0.00 ^a^ (0%)	0.00 ± 0.00 ^a^ (0%)
lungs	1.75 ± 0.53 ^a^ (87.5%)	0.63 ± 0.74 ^b^ (50%)	0.13 ± 0.35 ^b^ (12.5%)
thoracic airsacs	1.06 ± 0.86 ^a^ (75%)	0.31 ± 0.88 ^b^ (12.5%)	0.00 ± 0.00 ^b^ (0%)
abdominal airsacs	0.81 ± 0.84 ^a^ (75%)	0.31 ± 0.88 ^a,b^ (12.5%)	0.00 ± 0.00 ^b^ (0%)
pericardium	0.00 ± 0.00 ^a^ (0%)	0.00 ± 0.00 ^a^ (0%)	0.00 ± 0.00 ^a^ (0%)
spleen	0.69 ± 0.96 ^a^ (37.5%)	0.50 ± 0.76 ^a^ (37.5%)	0.00 ± 0.00 ^a^ (0%)
liver	0.88 ± 0.83 ^a^ (62.5%)	0.38 ± 0.74 ^a,b^ (25%)	0.00 ± 0.00 ^b^ (0%)
kidneys	0.25 ± 0.46 ^a^ (25%)	0.00 ± 0.00 ^a^ (0%)	0.00 ± 0.00 ^a^ (0%)
gut	0.00 ± 0.00 ^a^ (0%)	0.00 ± 0.00 ^a^ (0%)	0.00 ± 0.00 ^a^ (0%)
pectoral muscle	0.00 ± 0.00 ^a^ (0%)	0.00 ± 0.00 ^a^ (0%)	0.00 ± 0.00 ^a^ (0%)

**Table 4:** Histological pathology and inflammation scores for each organ, calculated by summing the scores from individual observations per bird, where each observation was scored on a scale from 0 (minimal to no change) to 5 (severe change). Upper airways were defined as the primary and secondary bronchi whereas lower airways were defined as tertiary bronchi and lung tissue. Values are presented as average score ± SD (% positive animals). Differences between treatment groups were determined with permutation tests and indicated with a different letter when significant.

	Tissue Organ	Control	mRNA LNPs	mRNA Galsomes
Pathology	Upper airways	1.25 ± 2.05 ^a^ (37.5%)	0.38 ± 1.06 ^a^ (12.5%)	0.00 ± 0.00 ^a^ (0%)
	Lower airways	0.00 ± 0.00 ^a^ (0%)	0.00 ± 0.00 ^a^ (0%)	0.00 ± 0.00 ^a^ (0%)
	Liver	1.25 ± 1.49 ^a^ (62.5%)	5.38 ± 5.34 ^a^ (87.5%)	1.25 ± 1.16 ^a^ (87.5%)
	Airsacs	0.50 ± 0.53 ^a^ (50%)	0.00 ± 0.00 ^a^ (0%)	0.50 ± 0.93 ^a^ (25%)
	Spleen	0.00 ± 0.00 ^a^ (0%)	0.00 ± 0.00 ^a^ (0%)	0.00 ± 0.00 ^a^ (0%)
	Pectoral muscle	0.00 ± 0.00 ^a^ (0%)	0.00 ± 0.00 ^a^ (0%)	0.00 ± 0.00 ^a^ (0%)
Inflammation	Upper airways	7.88 ± 5.87 ^a^ (87.5%)	5.38 ± 4.41 ^a,b^ (75%)	2.25 ± 2.76 ^b^ (50%)
	Lower airways	3.38 ± 1.41 ^a^ (87.5%)	3.13 ± 0.35 ^a^ (100%)	3.00 ± 0.53 ^a^ (100%)
	Liver	3.00 ± 0.76 ^a^ (100%)	1.88 ± 1.23 ^a^ (100%)	4.13 ± 3.18 ^a^ (100%)
	Airsacs	3.75 ± 3.33 ^a^ (75%)	1.43 ± 2.44 ^a^ (25%)	2.13 ± 2.30 ^a^ (62.5%)
	Spleen	0.88 ± 0.35 ^a^ (87.5%)	1.25 ± 0.46 ^a^ (100%)	1.25 ± 0.46 ^a^ (100%)
	Kidney	2.13 ± 0.35 ^a^ (100%)	2.88 ± 2.10 ^a^ (100%)	3.13 ± 1.81 ^a^ (100%)
	Pectoral muscle	0.00 ± 0.00 ^a^ (0%)	0.00 ± 0.00 ^a^ (0%)	0.00 ± 0.00 ^a^ (0%)

**Table 5:** Chlamydia presence scores in tissue organs. Values are presented as average score ± SD (% positive animals). Differences between treatment groups were determined with permutation tests and indicated with a different letter when significant.

Tissue Organ	Control	mRNA LNPs	mRNA Galsomes
lungs	2.57 ± 0.79 ^a^ (100%)	1.31 ± 0.88 ^b^ (87.5%)	0.88 ± 0.52 ^b^ (100%)
airsacs	1.94 ± 1.64 ^a^ (87.5%)	0.75 ± 0.54 ^a^ (87.5%)	1.38 ± 2.31 ^a^ (87.5%)
spleen	0.31 ± 0.37 ^a,b^ (50%)	0.50 ± 0.54 ^a^ (62.5%)	0.00 ± 0.00 ^b^ (0%)
liver	2.56 ± 1.82 ^a^ (100%)	1.94 ± 2.45 ^a,b^ (62.5%)	0.63 ± 0.70 ^b^ (50%)
kidneys	0.75 ± 1.04 ^a^ (50%)	0.50 ± 0.53 ^a^ (50%)	0.63 ± 0.74 ^a^ (50%)

## Data Availability

Data is contained within the article or Supplementary Material.

## Supplementary Materials

The following supporting information can be downloaded at: https://www.mdpi.com/article/10.3390/vaccines13020206/s1, Table S1: Scoring system for macroscopic lesions; Table S2: Histological findings scored by the pathologist. Scores were determined by multiplying the pathological grade assigned by the pathologist (blinded observations) for a specific pathologic observation by a weighting factor (derived from the frequency of animals within a group with a specific grade); Figure S1: Bleach gel electrophoresis. Lane 1: 10 kb MassRuler DNA ladder mix (Thermofisher); Lane 2: 1 µg un-digested plasmid picoZ::mRNA; Lane 3: 1 µg plasmid picoZ::mRNA digested with Sce-I (2595 bp); Lane 4: 1 µg cellulose purified mRNA::polyepitope (1386 bp); Figure S2: (a) pneumonic foci in the lung and opacity of thoracic airsacs of an infected animal; (b) Vascular injection in conjunctiva of an infected animal; (c) focal fibrin deposits in airsacs of an infected animal.
