# Supplementary material for: mRNA Galsomes Vaccine Protects Budgerigars Against Virulent Chlamydia psittaci Challenge
Source: Vaccines (Basel). 2025 Feb 19;13(2):206. doi: 10.3390/vaccines13020206 (PMC11861616; doi:10.3390/vaccines13020206)
Supplement: Supplementary file 1 [file vaccines-13-00206-s001.zip › vaccines-3456532-supplementary.pdf]

Supplementary Materials

Table S1. Scoring system for macroscopic lesions.

Tissue organ	Score 1	Score 2	Score 3
Conjunctiva	Congestion unilateral	Congestion bilateral	Petechiae
Conchae	Congestion bilateral	Congestion + mucus	Necrosis
Trachea	Slightly congested	Moderately congested	Severely congested
Lungs	Slightly congested	Severely congested	Grey foci
Thoracic airsacs	Diffuse opacity airsacculitis	Focal fibrin deposits	Severe fibrinous
Abdominal airsacs	Diffuse opacity airsacculitis	Focal fibrin deposits	Severe fibrinous
Pericardium	Serous pericarditis	Sero-fibrinous pericarditis	Fibrinous adhesive pericarditis
Spleen	Slightly enlarged	Moderately enlarged	Severely enlarged
Liver	Slightly enlarged	Moderately enlarged	Severely enlarged
Kidneys	Slightly congested	Moderately congested	Severely congested
Gut	Slightly congested	Moderately congested	Enteritis
Pectoral muscle	Slightly congested	Moderately congested	Severely congested

Table S2. Histological findings scored by the pathologist. Scores were determined by multiplying the pathological grade assigned by the pathologist (blinded observations) for a specific pathologic observation by a weighting factor (derived from the frequency of animals within a group with a specific grade).

		Histological score		
**Organ**	**Histological observation**	**Control**	**mRNA LNPs**	**mRNA Galsomes**
Upper airways	Formation of follicles and/or starry appearance indicative for stimulation	6	2	3
	Congestion	24	19	9
	Mononuclear cell infiltration, mostly lymphocytes	12	9	3
	Mixed cell infiltration, mononuclear cells and heterophils	3	2	0
	Infiltration subepithelial present	13	8	3
	Infiltration intraepithelial present	5	3	0
	Inflammatory cells in the bronchial lumen	0	0	0
	Mucoid content in the bronchial lumen	6	1	0
	Serofibrinous exudate in the bronchial lumen	0	0	0
	Fibrinous exudation in the bronchial lumen	0	0	0
	Hyperplasia/hypertrophy of the epithelium	4	2	0
Lower airways	Congestion	27	25	24
	Mononuclear cell infiltration, mostly lymphocytes	0	0	0
	Mixed cell infiltration, mononuclear cells and heterophils	0	0	0
	Infiltration subepithelial present	0	0	0
	Infiltration intraepithelial present	0	0	0
	Inflammatory cells in the bronchial lumen	0	0	0
	Mucoid content in the bronchial lumen	0	0	0
	Serofibrinous exudate in the bronchial lumen	0	0	0
	Fibrinous exudation in the bronchial lumen	0	0	0
	Hyperplasia/hypertrophy of the epithelium	0	0	0
Airsacs	Mononuclear cell infiltration, mostly lymphocytes	11	0	8
	Mixed cell infiltration, mononuclear cells and heterophils	7	4	0
	Infiltration subepithelial present	7	2	6

	Infiltration intraepithelial present	5	4	3
	Serofibrinous exudate	0	0	0
	Fibrinous exudation	0	0	0
	Hyperplasia/hypertrophy of the epithelium	4	0	4
	Necrotic material on the mucosa	0	0	0
**Liver**	Congestion	17	16	18
	Mononuclear cell infiltration, mostly lymphocytes	0	7	2
	Mixed cell infiltration, mononuclear cells and heterophils	0	7	4
	Lymphoid cell aggregates in the sinusoids	0	5	6
	Lymphoid cell aggregates in the portal areas	7	8	3
	Grainy degeneration to hydropic degeneration	10	15	10
**Spleen**	Congestion	7	8	8
	Mononuclear cell infiltration, mostly lymphocytes	0	0	0
	Mixed cell infiltration, mononuclear cells and heterophils	0	0	0
	Reticular cell hyperplasia: the reticular cells of the spleen are more prominent which can reflect lympho-depletion in the spleen	0	2	2
	Pronounced sheathed arteries	0	5	7
**Kidney**	Congestion	16	16	16
	Mononuclear cell infiltration, mostly lymphocytes	1	3	4
	Mixed cell infiltration, mononuclear cells and heterophils	0	0	0
	Infiltration subepithelial present	0	2	3
	Infiltration intraepithelial present	0	2	2
**Muscle**	Mononuclear cell infiltration, mostly lymphocytes	0	0	0
	Mixed cell infiltration, mononuclear cells and heterophils	0	0	0
	Necrosis	0	0	0

Figure S1. Bleach gel electrophoresis. Lane 1: 10 kb MassRuler DNA ladder mix (Thermofisher); Lane 2: 1 µg undigested plasmid picoZ::mRNA; Lane 3: 1 µg plasmid picoZ::mRNA digested with Sce-I (2595 bp); Lane 4: 1 µg cellulose purified mRNA::*polyepitope* (1386 bp).

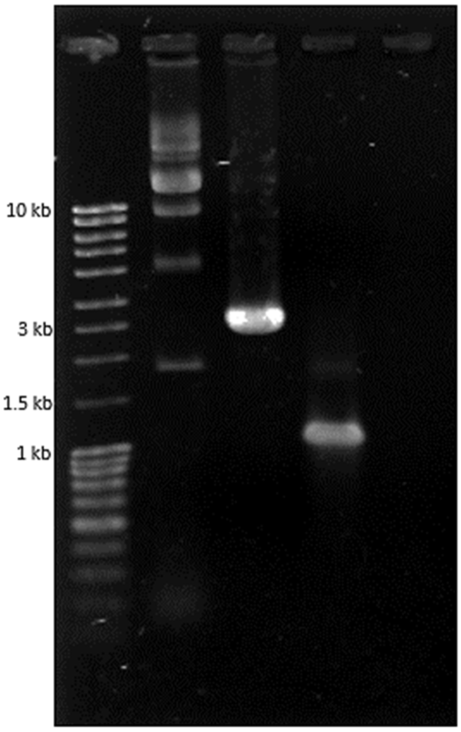

Figure S2. (**a**) pneumonic foci in the lung and opacity of thoracic airsacs of an infected animal; (**b**) Vascular injection in conjunctiva of an infected animal; (**c**) focal fibrin deposits in airsacs of an infected animal.

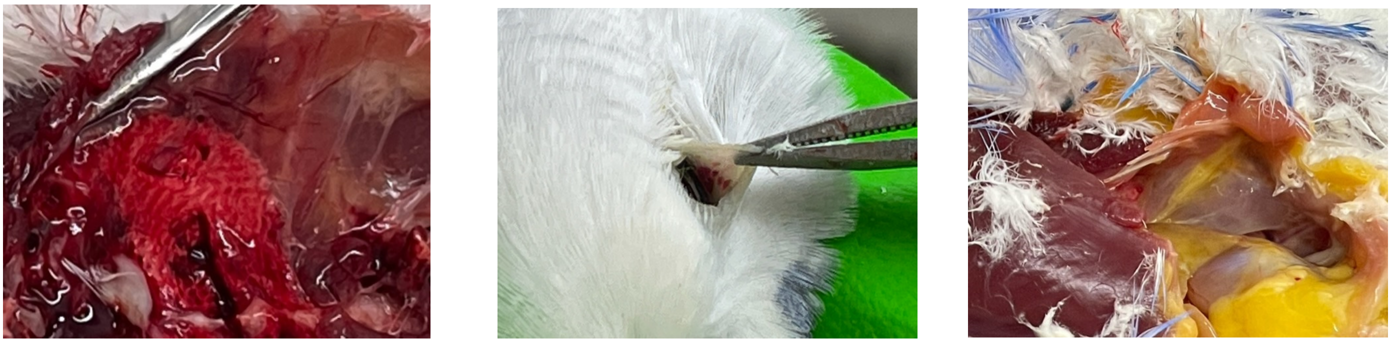

(**a**) (**b**) (**c**)
